# Oxidized phospholipids reduce ventilator-induced vascular leak and inflammation *in vivo*

**DOI:** 10.1186/cc6805

**Published:** 2008-01-24

**Authors:** Stephanie Nonas, Anna A Birukova, Panfeng Fu, Jungjie Xing, Santipongse Chatchavalvanich, Valery N Bochkov, Norbert Leitinger, Joe GN Garcia, Konstantin G Birukov

**Affiliations:** 1 Division of Pulmonary and Critical Care Medicine, Johns Hopkins University School of Medicine, Baltimore, MD 21224, USA; 2 Department of Medicine, University of Chicago, 5801 South Ellis St, Chicago, IL 60637, USA; 3 Department of Vascular Biology and Thrombosis Research, Medical University of Vienna, Schwarzspanierstrasse 17, 1090 Vienna, Austria; 4 Cardiovascular Research Center, University of Virginia, 415 Lane Rd, Charlottesville, VA 22908, USA

## Abstract

**Background:**

Mechanical ventilation at high tidal volume (HTV) may cause pulmonary capillary leakage and acute lung inflammation resulting in ventilator-induced lung injury. Besides blunting the Toll-like receptor-4-induced inflammatory cascade and lung dysfunction in a model of lipopolysaccharide-induced lung injury, oxidized 1-palmitoyl-2-arachidonoyl-sn-glycero-3-phosphorylcholine (OxPAPC) exerts direct barrier-protective effects on pulmonary endothelial cells *in vitro *via activation of the small GTPases Rac and Cdc42. To test the hypothesis that OxPAPC may attenuate lung inflammation and barrier disruption caused by pathologic lung distension, we used a rodent model of ventilator-induced lung injury and an *in vitro *model of pulmonary endothelial cells exposed to pathologic mechanochemical stimulation.

**Methods:**

Rats received a single intravenous injection of OxPAPC (1.5 mg/kg) followed by mechanical ventilation at low tidal volume (LTV) (7 mL/kg) or HTV (20 mL/kg). Bronchoalveolar lavage was performed and lung tissue was stained for histological analysis. *In vitro*, the effects of OxPAPC on endothelial barrier dysfunction and GTPase activation were assessed in cells exposed to thrombin and pathologic (18%) cyclic stretch.

**Results:**

HTV induced profound increases in bronchoalveolar lavage and tissue neutrophils and in lavage protein. Intravenous OxPAPC markedly attenuated HTV-induced protein and inflammatory cell accumulation in bronchoalveolar lavage fluid and lung tissue. *In vitro*, high-magnitude stretch enhanced thrombin-induced endothelial paracellular gap formation associated with Rho activation. These effects were dramatically attenuated by OxPAPC and were associated with OxPAPC-induced activation of Rac.

**Conclusion:**

OxPAPC exhibits protective effects in these models of ventilator-induced lung injury.

## Introduction

Acute lung injury (ALI) is a devastating clinical syndrome characterized by acute lung inflammation and vascular barrier disruption that affects more than 200,000 patients per year in the US and is associated with a mortality rate of 30% to 50% [1,2]. Mechanical ventilation, particularly with high tidal volumes (HTVs), can worsen or even cause *de novo *lung injury [3-5]. The landmark ARDSnet trial demonstrated a 22% decrease in mortality in acute respiratory distress syndrome (ARDS) with the use of low tidal volume (LTV) mechanical ventilation [6]. However, despite recent advances in LTV ventilatory strategies and a better understanding of the underlying inflammatory pathophysiology of ALI, there remain few effective treatments for this devastating illness. Meta-analyses of large-scale human trials have failed to show a mortality benefit from early high-dose corticosteroids, *N*-acetylcysteine, surfactant, or prostaglandin E_1 _despite promising preclinical studies [7]. Thus, ALI and ventilator-induced lung injury (VILI) continue to present a significant clinical challenge, and novel treatments aimed at reducing vascular leak and acute inflammation in lung injury are needed.

Cell-membrane phospholipids and phospholipids present in circulating lipoproteins may undergo oxidation by lipoxygenases or reactive oxygen and nitrogen species as a result of VILI, trauma, or septic inflammation [8-13]. One of the major plasma membrane phospholipids is 1-palmitoyl-2-arachidonoyl-sn-glycero-3-phosphorylcholine (PAPC), which upon oxidation (OxPAPC) may propagate chronic vascular inflammatory processes involved in atherogenesis [14-17] but also exhibit potent anti-inflammatory effects in acute settings [8-13]. Administration of a mixture of lipopolysaccharide (LPS) and OxPAPC decreases inflammatory cell recruitment and cytokine production in the lungs [18] and even protects against LPS-mediated lethal shock [19]. We recently demonstrated that intravenously administered OxPAPC protects against tissue inflammation, lung vascular barrier dysfunction, and inflammatory cytokine production caused by aerosolized LPS [20]. The observation that intravenous injection of OxPAPC significantly attenuated leukocyte extravasation and decreased bronchoalveolar lavage (BAL) protein content induced by intratracheal administration of LPS suggested that the *in vivo *protective effect of OxPAPC may be associated, in part, with its direct effects on the vascular endothelial barrier.

Previously, we described potent Rac-dependent barrier-protective effects of oxidized phospholipids on cultured pulmonary endothelial cells (ECs) and identified the critical role of cyclopentenone-containing oxidized modifications of arachidonoyl moiety and polar head groups (choline and serine) in the mediation of the OxPAPC effects [21,22]. Our published data demonstrate the ability of barrier-protective oxidized phospholipids to attenuate thrombin-induced stress fiber and paracellular gap formation, Rho activation, myosin light chain phosphorylation, and hyperpermeability. Furthermore, barrier-protective effects of OxPAPC in the model of thrombin-induced EC barrier dysfunction are associated with stimulation of Rac signaling critical for EC barrier recovery [21,23,24].

In this study, we used rodent models of VILI and pulmonary ECs exposed to physiologic and pathologic levels of cyclic stretch (CS) and thrombin stimulation to test the hypotheses that vascular leak caused by mechanical ventilation at HTVs involves the Rho pathway of endothelial barrier dysfunction and that OxPAPC may attenuate Rho activation induced by VILI-associated pathologic mechanochemical stimulation via Rac-dependent mechanisms. Selected parts of this study were presented at the American Thoracic Society International Conference in San Diego, California, 20 to 25 May 2006.

## Materials and methods

### Animal studies

Adult male Brown Norway rats (250 to 350 g) (Charles River Laboratories, Inc., Wilmington, MA, USA) or adult male C57BL/6J mice (8 to 10 weeks old with an average weight of 20 to 25 g) (The Jackson Laboratory, Bar Harbor, ME, USA) were anesthetized with an intraperitoneal injection of ketamine (75 mg/kg) and acepromazine (1.5 mg/kg). All rat studies were performed using 2-hour mechanical ventilation. Tracheotomy was performed and the trachea was cannulated with a 14-guage intravenous catheter, which was tied into place to prevent air leak. Rats were assigned to either HTV (20 mL/kg) or LTV (7 mL/kg) mechanical ventilation at 85 breaths per minute and 0 positive end-expiratory pressure (PEEP) for 2 hours. Arterial blood pressure and pH were monitored via a carotid artery catheter at 30-minute intervals. External dead space in the HTV group allowed the maintenance of blood pH of 7.30 to 7.44. Intravenous fluid boluses of phosphate-buffered saline (PBS) were given to maintain a mean arterial pressure of greater than 65 mm Hg. Rats were randomly assigned to concurrently receive an intravenous bolus of sterile PBS or OxPAPC (1.5 mg/kg) via the jugular vein at the initiation of mechanical ventilation. At the end of each experiment, rats were killed by exsanguination under anesthesia, and BAL was performed on the left lung using 3 mL of sterile PBS. BAL inflammatory cell counting was performed using a standard hemacytometer technique. Differential cell counts were performed on Diff-Quick-stained (Baxter Diagnostics, McGaw Park, IL, USA) slides with a minimum of 300 cells per slide. The BAL protein concentration was determined by a modified Lowry colorimetric assay using a Bio-Rad DC protein assay kit (Bio-Rad Laboratories, Inc., Hercules, CA, USA). In subsequent experiments, mechanical ventilation of mice was performed for 4 hours as we [25] and others [26] have previously described. Mice were treated intravenously with OxPAPC (1.5 mg/kg), oxidation-resistant phospholipid (di-myristoyl-sn-glycero-3-phosphorylcholine [DMPC]) (1.5 mg/kg), Rho inhibitor Y27632 (10 mg/kg), or thrombin signaling peptide TRAP-6 (thrombin receptor activating peptide-6) (3 × 10^-7 ^mol/mouse) followed by HTV or LTV (30 or 7 mL/kg, respectively, at 75 breaths per minute and 0 PEEP for 4 hours). Control animals were anesthetized and allowed to breathe spontaneously. At sacrifice, BAL of both lungs was performed with 1 mL of sterile Hanks' balanced salt solution for measurement of inflammatory cells and protein. All animal experiments were approved by the Institutional Animal Care and Use Committee at Johns Hopkins University and the University of Chicago. The animals were housed in pathogen-free conditions in the Johns Hopkins Asthma and Allergy Center and the University of Chicago Animal Care Facilities, where they were cared for in accordance with institutional and National Institutes of Health (Bethesda, MD, USA) guidelines.

### Histological assessment for lung injury

At sacrifice, the lungs were harvested without lavage collection and fixed in 4% paraformaldehyde. After fixation, the lungs were embedded in paraffin, cut into 4-μm sections, and stained with hematoxylin and eosin. Sections were evaluated at × 400 magnification.

### Measurement of Evans blue accumulation

Measurement of Evans blue accumulation in the lung tissue was performed by spectrofluorimetric analysis of lung tissue lysates according to the protocol described previously [27].

### Reagents and cell culture

PAPC was obtained from Sigma-Aldrich (St. Louis, MO, USA) and oxidized by exposure of dry lipid to air for 72 hours. The extent of oxidation was monitored by positive-ion electrospray mass spectrometry as described previously [20,21,28]. Human pulmonary macro- and microvascular ECs were obtained from Lonza Inc (Allendale, NJ, USA), maintained according to the vendor's protocol, and used at passages 5 to 8 for CS experiments as previously described [29,30]. Human thrombin was obtained from Sigma-Aldrich. RhoA and Rac1 antibodies were obtained from Santa Cruz Biotechnology, Inc. (Santa Cruz, CA, USA).

### Cell culture under cyclic stretch

All CS experiments were performed using an FX-4000T Flexercell Tension Plus system (Flexcell International Corporation, Hillsborough, NC, USA) equipped with a 25-mm BioFlex Loading Station as previously described [29,30]. Experiments were performed in the presence of culture medium containing 2% fetal bovine serum. Briefly, ECs were seeded at standard densities (8 × 10^5 ^cells per well) onto collagen I-coated flexible-bottom BioFlex plates. After 48 hours of culture, each plate received fresh medium, was mounted onto the Flexercell system, and was exposed for 2 hours to either low-magnitude (5% elongation) or high-magnitude (18% elongation) CS to recapitulate the mechanical stresses experienced by the alveolar endothelium during normal respiration and HTV mechanical ventilation, respectively [29,31,32]. At 2 hours, a subset of plates were treated with OxPAPC (20 μg/mL) for 15 minutes followed by treatment with thrombin (0.5 U/mL) and incubation for 15, 30, or 50 minutes with continuous exposure to CS. Control BioFlex plates with static EC culture treated with OxPAPC and/or thrombin were placed in the same cell culture incubator. At the end of experiment, cell lysates were collected for Rac and Rho activation assays, or CS-exposed endothelial monolayers were fixed with 3.7% formaldehyde and used for immunofluorescence staining as previously described [21,33].

### Rho and Rac activation assays

Rho and Rac activation assays were performed using commercially available assay kits purchased from Upstate Biotechnology (Lake Placid, NY, USA) as we have previously described [21,33].

### Measurement of transendothelial electrical resistance

The cellular barrier properties were analyzed by measurement of transendothelial electrical resistance across confluent human pulmonary artery and human lung microvascular endothelial monolayers using an electrical cell-substrate impedance sensing system (Applied BioPhysics, Inc., Troy, NY, USA) as previously described [21,33,34].

### Immunofluorescence staining

After exposure to CS and agonist stimulation, ECs were subjected to immunofluorescence staining to visualize actin filaments as previously described [21,33].

### Statistical methods

All *in vivo *data are presented as mean ± standard deviation. Group comparisons were evaluated by the analysis of variance test with *post hoc *Newman-Keuls multiple comparison test. *P *values of less than 0.05 were considered statistically significant.

## Results

### Effects of OxPAPC on ventilator-induced lung inflammation and barrier dysfunction

**Figure 1:**
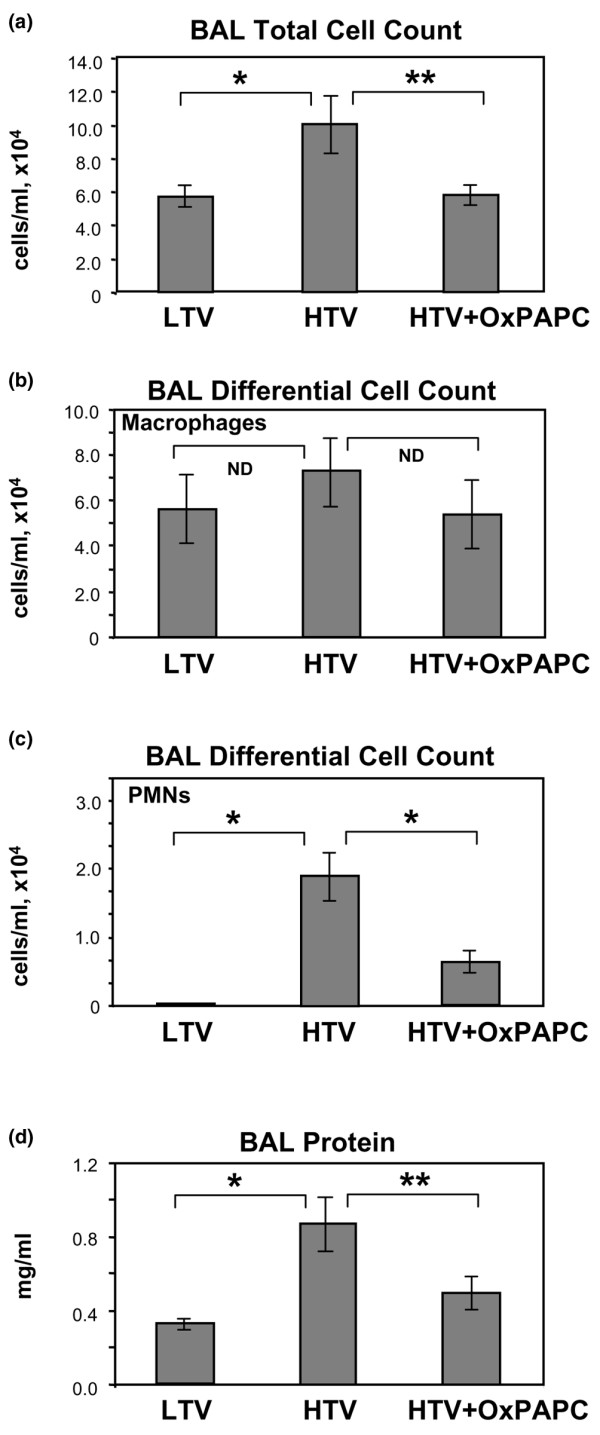
Effects of OxPAPC on inflammatory cell recruitment in bronchoalveolar lavage (BAL) fluid of rats exposed to high tidal volume (HTV). HTV (20 mL/kg, 2 hours) induced a marked increase in BAL total cell count **(a) **and macrophages and neutrophils **(b) **compared with low tidal volume (LTV) controls. Intravenous OxPAPC (1.5 mg/kg) markedly attenuated this response, reducing inflammatory cells to control levels and significantly reducing neutrophil influx. *p < 0.05 versus LTV, **p < 0.05 versus HTV (*n *= 5 to 6 per group). **(c) **BAL protein concentration was assessed as a measure of vascular barrier disruption following 2 hours of mechanical ventilation with LTV or HTV. Intravenous OxPAPC (1.5 mg/kg) significantly reduced the pronounced increase in BAL protein induced by HTV mechanical ventilation (*p < 0.01 versus LTV, **p < 0.05 versus HTV). ND, no difference; OxPAPC, oxidized 1-palmitoyl-2-arachidonoyl-sn-glycero-3-phosphorylcholine; PMN, polymorphonuclear leukocyte.

**Figure 2:**
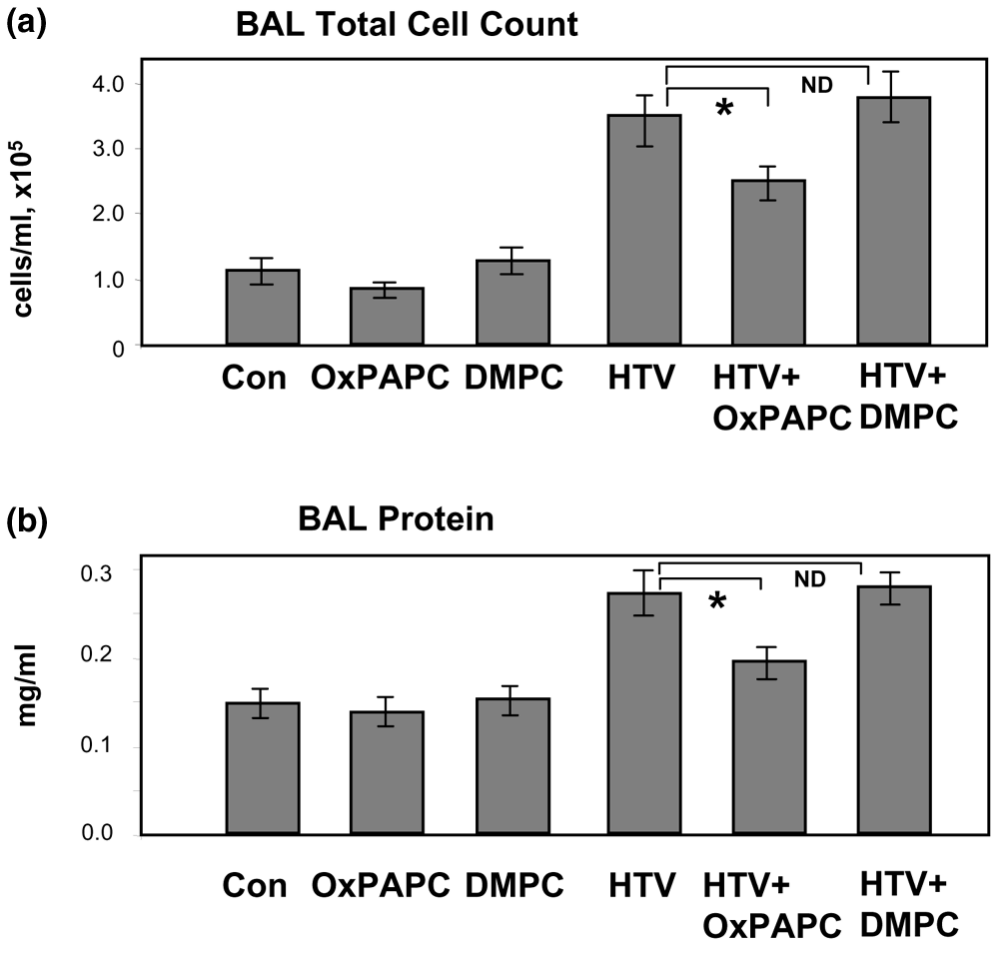
Effects of OxPAPC and DMPC on inflammatory cell recruitment in bronchoalveolar lavage (BAL) fluid of mice exposed to high tidal volume (HTV). HTV (30 mL/kg, 4 hours) induced a dramatic increase in BAL total cell count **(a) **and protein content **(b)**, which was markedly attenuated by intravenous injection of OxPAPC (1.5 mg/kg) but not DMPC (1.5 mg/kg). There were no significant differences in cell counts and protein content between animals treated with vehicle, OxPAPC, or DMPC alone. *p < 0.05 (*n *= 6 to 9 per group). Con, control; DMPC, di-myristoyl-sn-glycero-3-phosphorylcholine; ND, no difference; OxPAPC, oxidized 1-palmitoyl-2-arachidonoyl-sn-glycero-3-phosphorylcholine.

We evaluated the effects of intravenously administered OxPAPC on the parameters of lung inflammation and barrier dysfunction in rats exposed to mechanical ventilation at HTV (20 mL/kg) compared with control rats exposed to 'protective' LTV mechanical ventilation (7 mL/kg) [35-37]. In our previous studies, we have determined the range of OxPAPC doses (1.5 to 3.0 mg/kg) that provided the optimal barrier protection *in vivo *and demonstrated that at these doses OxPAPC alone did not change total cell count, neutrophil count, or protein content in the BAL of uninjured control animals [20]. Rats received a single intravenous dose of OxPAPC (1.5 mg/kg) or sterile PBS at the onset of HTV or LTV mechanical ventilation. At 2 hours, BAL and tissue harvesting were performed as described above. HTV induced an increase in BAL inflammatory cell count in comparison with LTV controls (9.92 × 10^4 ^± 1.79 versus 5.83 × 10^4 ^± 0.72 cells per milliliter in LTV controls) (Figure 1a). This effect was due mainly to an influx of polymorphonuclear leukocytes (PMNs) (Figure 1b, bottom), and OxPAPC markedly attenuated both total BAL cell count (5.89 × 10^4 ^± 0.55 versus 9.92 × 10^4 ^± 1.79 cells per milliliter in HTV) and BAL PMNs (1.57 ± 0.32 × 10^4 ^versus 3.15 ± 0.86 × 10^3 ^cells per milliliter in HTV). Statistical analysis of BAL macrophages (Figure 1b, top) showed that, despite a small trend toward increased alveolar macrophages in the HTV group compared with LTV controls and HTV + OxPAPC-treated animals, there were no statistically significant differences in macrophage counts among the three groups. Likewise, HTV caused significant barrier disruption, inducing a 1.7-fold increase in BAL protein compared with LTV controls (0.873 ± 0.136 versus 0.325 ± 0.038 mg/mL in control). This effect was significantly attenuated by a single intravenous injection of OxPAPC (0.500 ± 0.092 versus 0.873 ± 0.136 mg/mL in HTV alone) (Figure 1c). *T *test comparison of non-ventilated controls with LTV controls showed no statistically significant differences in BAL inflammatory cells or protein between the two groups (data not shown). Because PAPC is subject to *in vivo *oxidation to OxPAPC, analyses of BAL protein and cell count were performed in an additional series of experiments in a mouse model of VILI using the oxidation-resistant PAPC analog, DMPC. DMPC had no effect on either BAL cell count or protein in control animals and did not protect against HTV-induced cell and protein accumulation in the BAL (Figure 2). These results are also consistent with the lack of barrier-protective effects by DMPC in EC cultures, as we have previously described [21]. As in our previous study, OxPAPC had no significant effect on BAL cell count or protein content in uninjured control animals (Figure 2).

**Figure 3:**
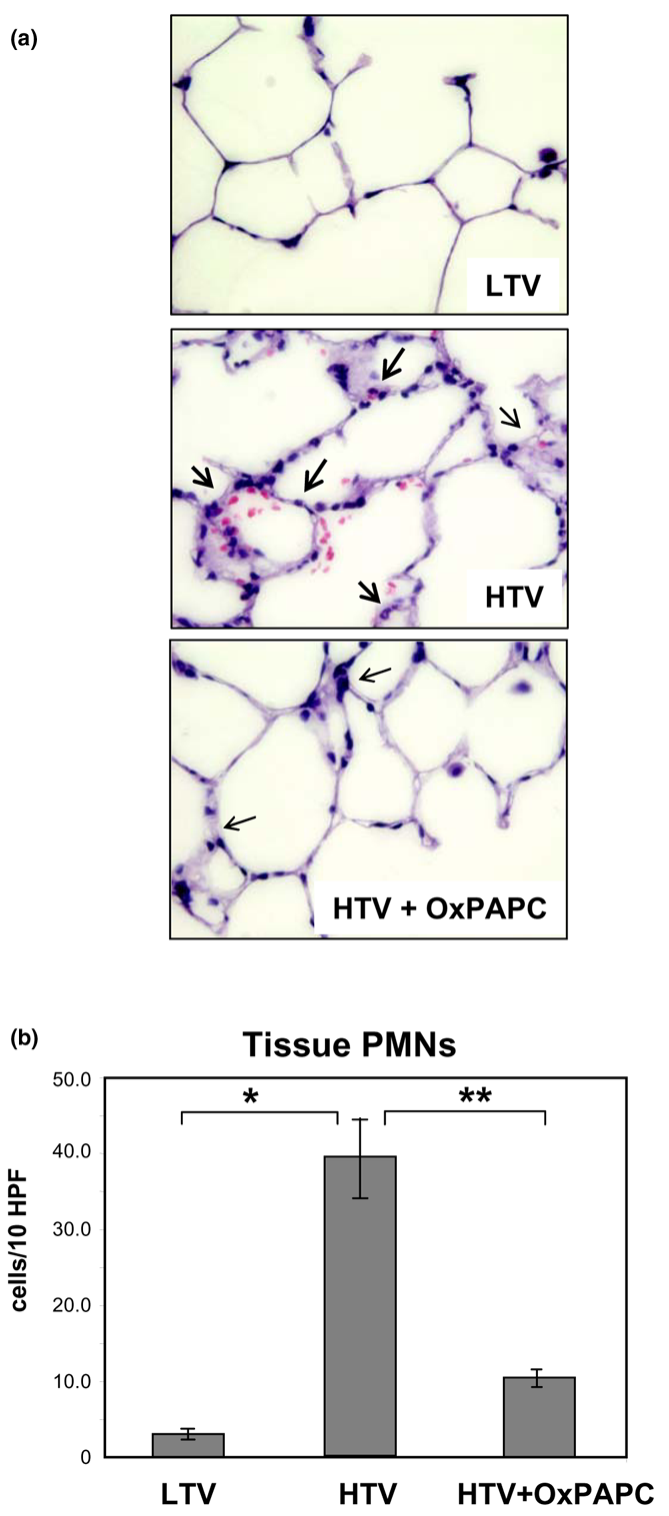
Histological assessment of the effect of OxPAPC on ventilator-induced lung injury. Whole lungs (4 to 6 animals from each experimental group) were agarose-inflated *in situ*, fixed in 10% formalin, and used for histologic evaluation by hematoxylin and eosin staining as described in Materials and methods. Histological analysis of lung tissue (×40 magnification) **(a) **and quantitative analysis of lung tissue neutrophil count **(b) **obtained from rats exposed to high tidal volume (HTV) mechanical ventilation demonstrate a neutrophilic inflammation and areas of alveolar hemorrhage, which were attenuated by co-treatment with intravenous OxPAPC. For tissue polymorphonuclear leukocyte (PMN) counts, 10 fields per slide were counted for *n *= 4 animals per experimental group. *p < 0.01 versus low tidal volume (LTV), **p < 0.05 versus HTV (*n *= 4 to 6 per group). HPF, high power microscopic field; OxPAPC, oxidized 1-palmitoyl-2-arachidonoyl-sn-glycero-3-phosphorylcholine.

Histological analysis of paraffin-embedded rat lung sections stained with hematoxylin and eosin revealed parenchymal inflammatory cell recruitment (neutrophils noted with arrows) and areas of alveolar hemorrhage indicative of vascular disruption with HTV ventilation that was attenuated with OxPAPC (Figure 3a). Quantitative analysis of acute tissue inflammation revealed a 10-fold increase in tissue PMNs with HTV ventilation (39.94 ± 12.4 per 10 high power microscopic fields [HPF] versus 3.33 ± 1.36 per 10 HPF in LTV controls) that was significantly reduced by OxPAPC (10.08 ± 2.75 per 10 HPF versus 39.94 ± 12.4 per 10 HPF in HTV) (Figure 3b). Injection of non-oxidized PAPC (1.5 mg/kg) was without effect (data not shown).

**Figure 4:**
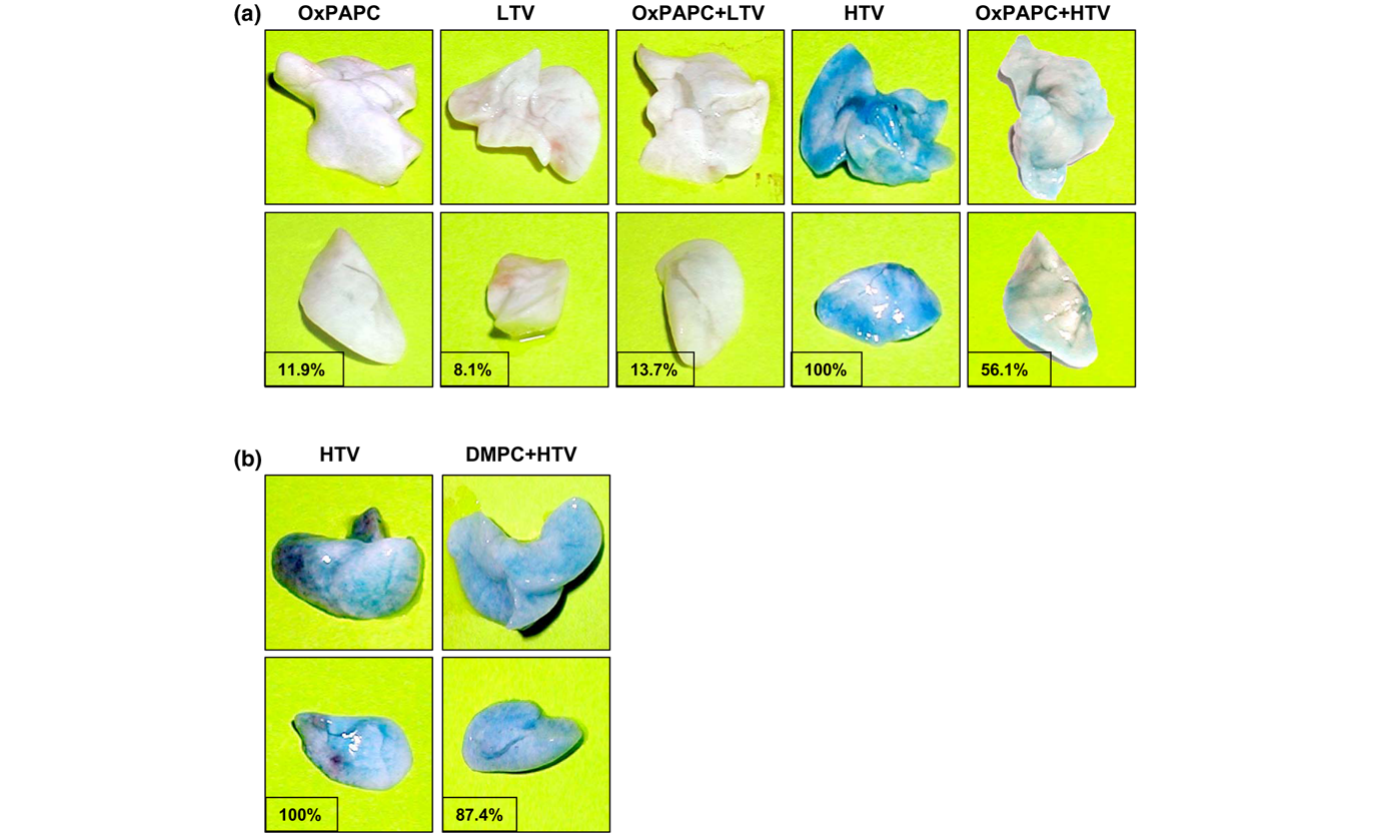
Effects of OxPAPC on high tidal volume (HTV)-induced lung vascular leak. Mice were treated with OxPAPC (1.5 mg/kg, intravenous) or DMPC (1.5 mg/kg, intravenous) followed by mechanical ventilation at low tidal volumes (LTVs) (7 mL/kg) or HTVs (30 mL/kg) for 4 hours. Effects of phospholipids on the HTV-induced vascular leak were assessed by measurements of Evans blue leakage into the lung tissue. HTV, but not LTV, induced Evans blue leakage from the vascular space into surrounding lung tissue, which was dramatically attenuated by OxPAPC **(a)**, but not by DMPC **(b)**, pretreatment. The results are representative of three independent experiments. Insets depict the quantitative analysis of Evans blue-labeled albumin extravasation in the shown lung preparations, which was performed by spectrophotometric analysis of Evans blue extracted from the lung tissue samples as described in Materials and methods. Evans blue accumulation in the lungs from HTV-exposed animals (122 ± 12 μg/g wet weight lung tissue) was taken as 100%. The results are representative of three independent experiments. DMPC, di-myristoyl-sn-glycero-3-phosphorylcholine; OxPAPC, oxidized 1-palmitoyl-2-arachidonoyl-sn-glycero-3-phosphorylcholine.

The protective effects of OxPAPC against vascular leak were further assessed by measurement of Evans blue leakage into the lung tissue. HTV induced noticeable Evans blue leakage from the vascular space into the lung parenchyma, which was significantly decreased by OxPAPC pretreatment (Figure 4a). Importantly, the oxidation-resistant PAPC analog DMPC did not significantly reduce Evans blue accumulation in the tissue (Figure 4b). Thus, our data clearly demonstrate protective effects of OxPAPC in both rat and mouse models of ALI induced by mechanical ventilation at HTV.

### Involvement of Rho pathway in ventilator-induced lung inflammation and barrier dysfunction

**Figure 5:**
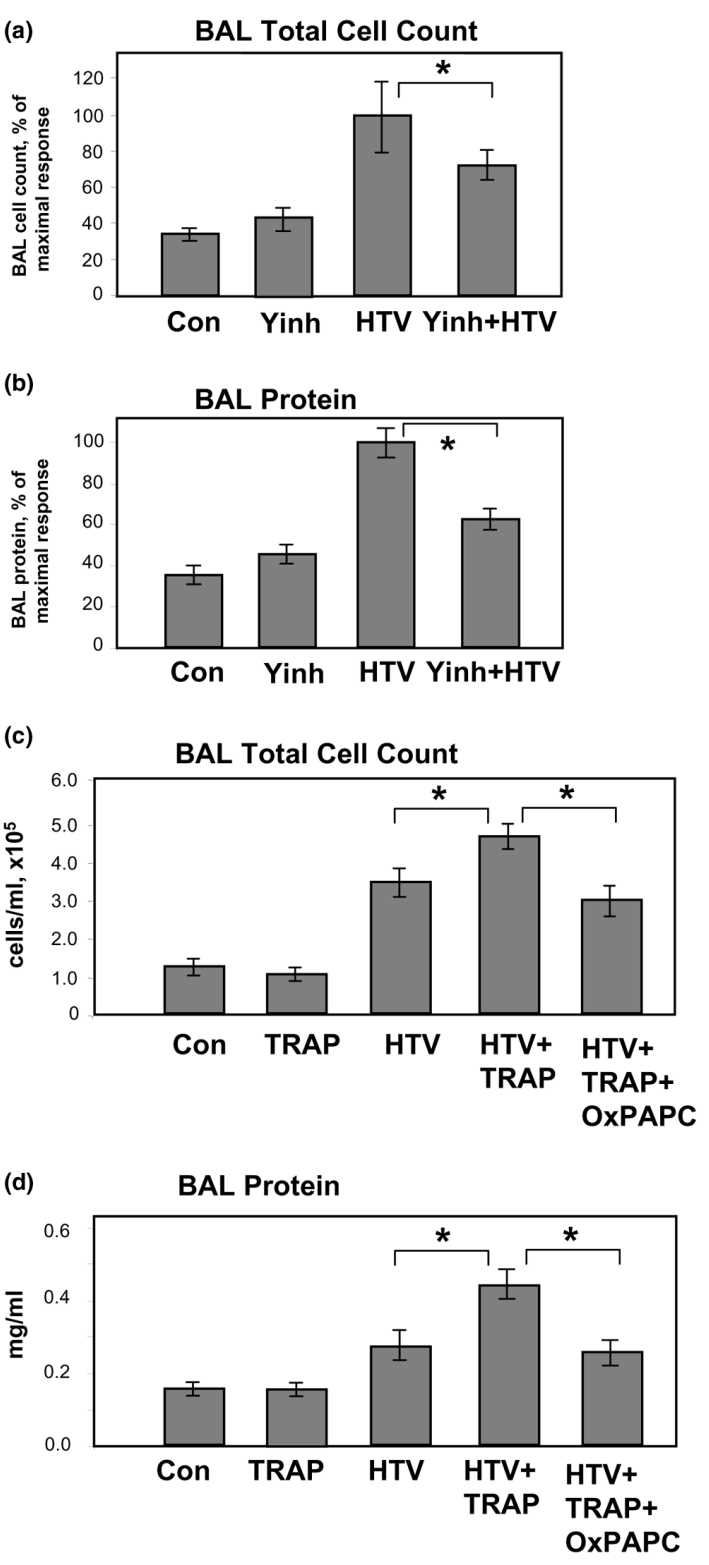
Effects of Rho kinase inhibition on severity of high tidal volume (HTV)-induced lung injury. Mice received a single dose of Rho kinase inhibitor Y27632 (10 mg/kg, intraperitoneal) or TRAP-6 (3 × 10^-7 ^mol/mouse, intravenous) followed by mechanical ventilation (30 mL/kg, 4 hours) with or without OxPAPC injection (1.5 mg/kg, intravenous). Inhibition of the Rho pathway markedly attenuated HTV-induced bronchoalveolar lavage (BAL) cell count and protein content **(a,b)**. TRAP-6 further enhanced HTV-induced increases in BAL cell count and protein content **(c,d)**, whereas OxPAPC significantly reduced these effects. *p < 0.05 (*n *= 4 to 8 per group). Con, control; OxPAPC, oxidized 1-palmitoyl-2-arachidonoyl-sn-glycero-3-phosphorylcholine; TRAP-6, thrombin receptor activating peptide-6; Yinh, Y27632.

In the following experiments, we investigated a role of Rho-dependent signaling in lung injury induced by mechanical ventilation. Pharmacologic inhibition of Rho-associated kinase by Y-27632 markedly attenuated HTV-induced increases in lung BAL cell count and protein content in our murine VILI model (Figure 5a,b), suggesting the involvement of Rho signaling in the lung dysfunction caused by mechanical stress. We have previously described attenuation of thrombin-induced endothelial barrier dysfunction by OxPAPC via Rac-dependent suppression of Rho activity [21,22]. Taken together, these results strongly suggest Rac-Rho crosstalk as the mechanism underlying protective effects of OxPAPC in the model of VILI.

It is important to note that disturbances in coagulation and fibrinolysis have been clearly demonstrated in patients with ALI/ARDS. Recent reports also suggest that mechanical ventilation may lead to or aggravate pulmonary coagulopathy [38]. Because thrombin is known to activate Rho both *in vivo *and *in vitro*, increased thrombin levels may become a considerable factor contributing to the Rho-mediated vascular endothelial barrier dysfunction caused by HTV mechanical ventilation. Because the *in vivo *use of thrombin is limited due to significant intravascular thrombosis, we performed additional experiments using thrombin-derived non-thrombogenic PAR-1 (protease-activated receptor-1) receptor ligand TRAP-6 in our murine VILI model. Mice were given a single dose of intravenous TRAP-6 (3 × 10^-7 ^mol/mouse) followed by 4 hours of HTV mechanical ventilation. Measurements of BAL protein concentration and cell count revealed that TRAP-6 exacerbated HTV-induced lung dysfunction, inducing a 36% ± 6.7% increase in BAL inflammatory cells and a 62% ± 9.2% increase in BAL protein compared with animals treated only with HTV. Notably, OxPAPC, but not its oxidation-resistant analog DMPC (data not shown), significantly reduced these parameters of lung injury in TRAP-6-treated animals (Figure 5c,d).

### Effects of OxPAPC on monolayer recovery in human pulmonary endothelial cells exposed to cyclic stretch and thrombin

**Figure 6:**
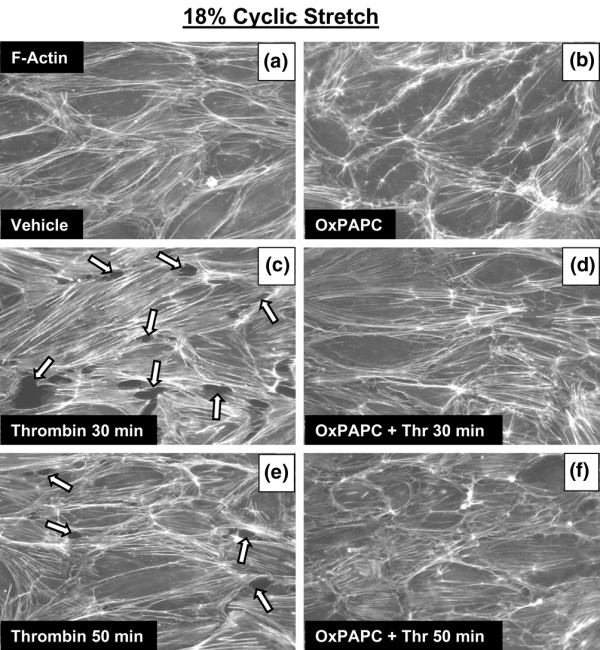
Cells subjected to pathologic cyclic stretch (CS) (18%, 2 hours) were pretreated with vehicle **(a) **or OxPAPC (20 μg/mL) **(b) **followed by thrombin (0.5 U/mL) stimulation for 30 **(c,d) **or 50 **(e,f) **minutes. F-actin was visualized by immunofluorescence staining with Texas-Red phalloidin. Cells subjected to CS and thrombins (30 or 50 minutes) demonstrate barrier disruption, with the formation of transcellular actin stress fibers resulting in increased tension, cellular contraction, and paracellular gap formation (arrows). OxPAPC enhanced monolayer integrity and peripheral actin cytoskeletal rearrangement in ECs exposed to 18% CS alone and dramatically attenuated thrombin-induced gap formation and disruption of monolayer integrity and accelerated EC barrier recovery. Representative results from three independent experiments are shown. Two wells from each experiment were observed for each stimulation. OxPAPC, oxidized 1-palmitoyl-2-arachidonoyl-sn-glycero-3-phosphorylcholine; Thr, thrombin.

Thrombin stimulation of pulmonary ECs exposed to pathologically relevant levels of CS *in vitro *was used to reproduce a 'double-hit' model of VILI and lung vascular dysfunction, combining excessive levels of mechanical ventilation with an edemagenic agent (thrombin) known to activate Rho signaling [33,39-42]. Using this model, we evaluated the protective effects of OxPAPC. We have previously shown that pathologic CS (18% elongation) enhances endothelial disruption induced by the edemagenic agonist thrombin as indicated by pronounced paracellular gap formation and activation of actomyosin contraction governed by increases in myosin light chain phosphorylation [23]. Human pulmonary ECs were exposed to pathologic 18% CS for 2 hours and treated with OxPAPC (20 μg/mL, 15 minutes) or vehicle followed by stimulation with thrombin (0.5 U/mL) with continuing CS (Figure 6). Cells exposed to OxPAPC alone during CS revealed enhanced monolayer integrity, with increased peripheral F-actin staining and a reduced number of central actin stress fibers (Figure 6b). PAPC alone affects neither F-actin remodeling nor small GTPase activity [21]. Consistent with our recent studies [23], thrombin treatment of pulmonary ECs exposed to 18% CS induced rapid barrier disruption with dramatic paracellular gap formation and enhanced stress fiber formation (Figure 6c) with partial recovery by 50 minutes (Figure 6e). Pretreatment with OxPAPC dramatically attenuated the paracellular gap formation in ECs exposed to 18% CS at 30 minutes of thrombin treatment and completely restored EC cytoskeletal organization and monolayer integrity at 50 minutes (Figure 6d,f). At the 30-minute time point, monolayer recovery in the OxPAPC-treated cells was almost complete (Figure 6d) in comparison with the delayed monolayer recovery of the cells without OxPAPC treatment (Figure 6c,e). Similar to the 30-minute time point, OxPAPC treatment diminished stress fiber and gap formation in the endothelial monolayers exposed to 18% CS and thrombin for 15 minutes (data not shown). These data demonstrate that OxPAPC enhances pulmonary EC monolayer integrity and peripheral actin cytoskeletal rearrangement in CS-preconditioned pulmonary ECs without thrombin treatment, dramatically attenuates paracellular gap formation in cells exposed to 18% CS and thrombin, and accelerates EC barrier recovery after thrombin challenge.

**Figure 7:**
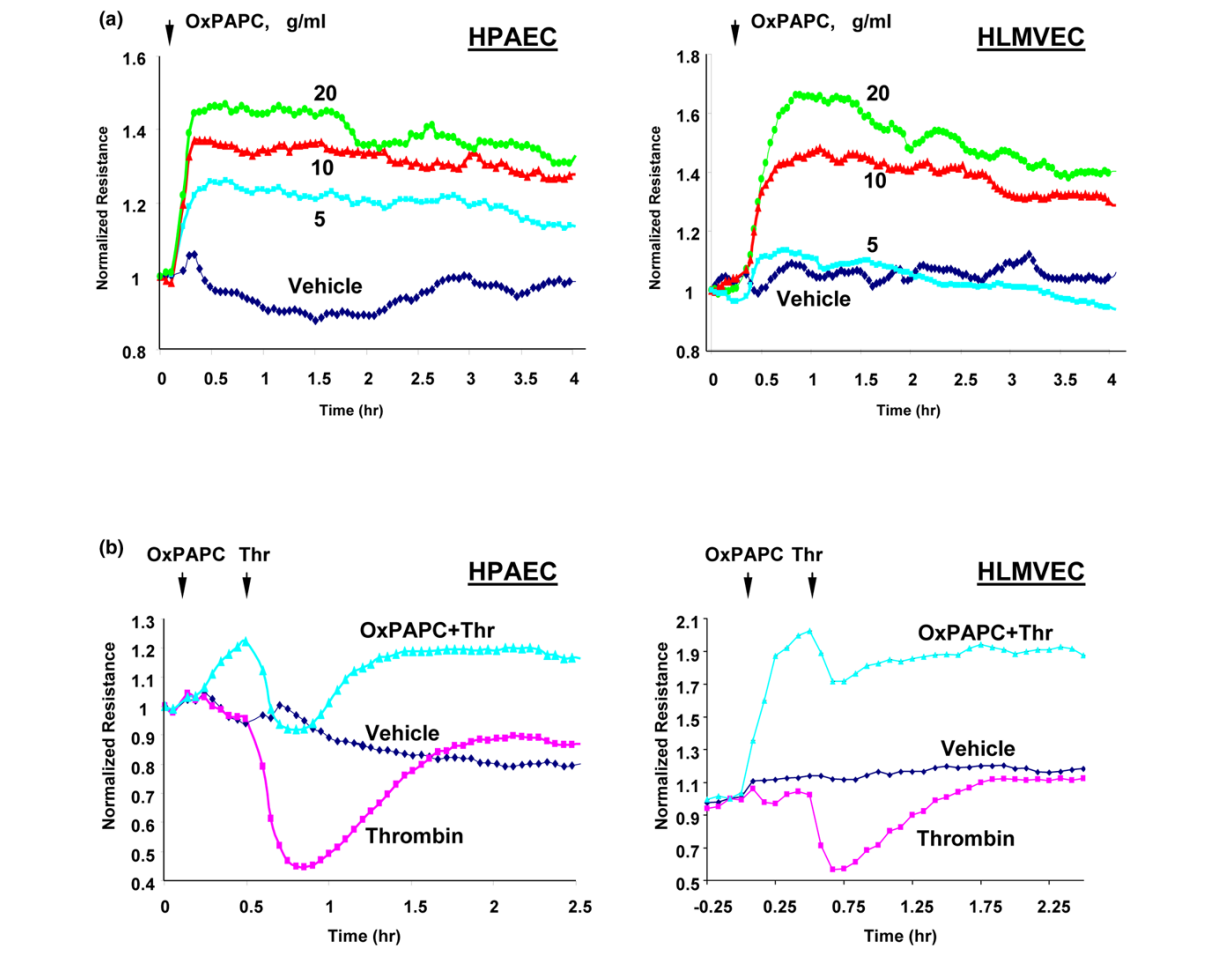
Effects OxPAPC on barrier properties in macro- and microvascular pulmonary endothelial cells (ECs). Human pulmonary artery (HPAEC) or microvascular (HLMVEC) ECs were plated on gold microelectrodes to measure transendothelial electrical resistance (TER) and were cultured to confluence. Growth medium was replaced with serum-free Opti-MEM (Invitrogen Corporation, Carlsbad, CA, USA). After equilibration and stabilization, measurements of TER were performed. At the time indicated by the arrow, ECs were treated with various concentrations of OxPAPC **(a) **or cells were pretreated with OxPAPC (20 μg/mL) followed by thrombin (0.5 U/mL) stimulation (marked by second arrow) **(b)**. Results are representative of three to six independent experiments. OxPAPC, oxidized 1-palmitoyl-2-arachidonoyl-sn-glycero-3-phosphorylcholine; Thr, thrombin.

Because the site of transvascular flux in the lungs is the microvasculature, and phenotypic differences between macro- and microvascular endothelium are well recognized, we next used human lung microvascular ECs to further characterize the effects of oxidized phospholipids. Our results demonstrate that, as with human pulmonary artery ECs (HPAECs), OxPAPC increased baseline transendothelial electrical resistance in human lung microvascular ECs in a dose-dependent manner (Figure 7a). Furthermore, OxPAPC was protective against thrombin-induced permeability in both macro- and microvascular ECs (Figure 7b). Interestingly, macro- and microvascular ECs exhibited different sensitivities to OxPAPC and, in microvascular EC OxPAPC, induced a greater barrier-protective effect than in macrovascular ECs.

### Effects of OxPAPC on Rac and Rho activation following cyclic stretch and thrombin stimulation

**Figure 8:**
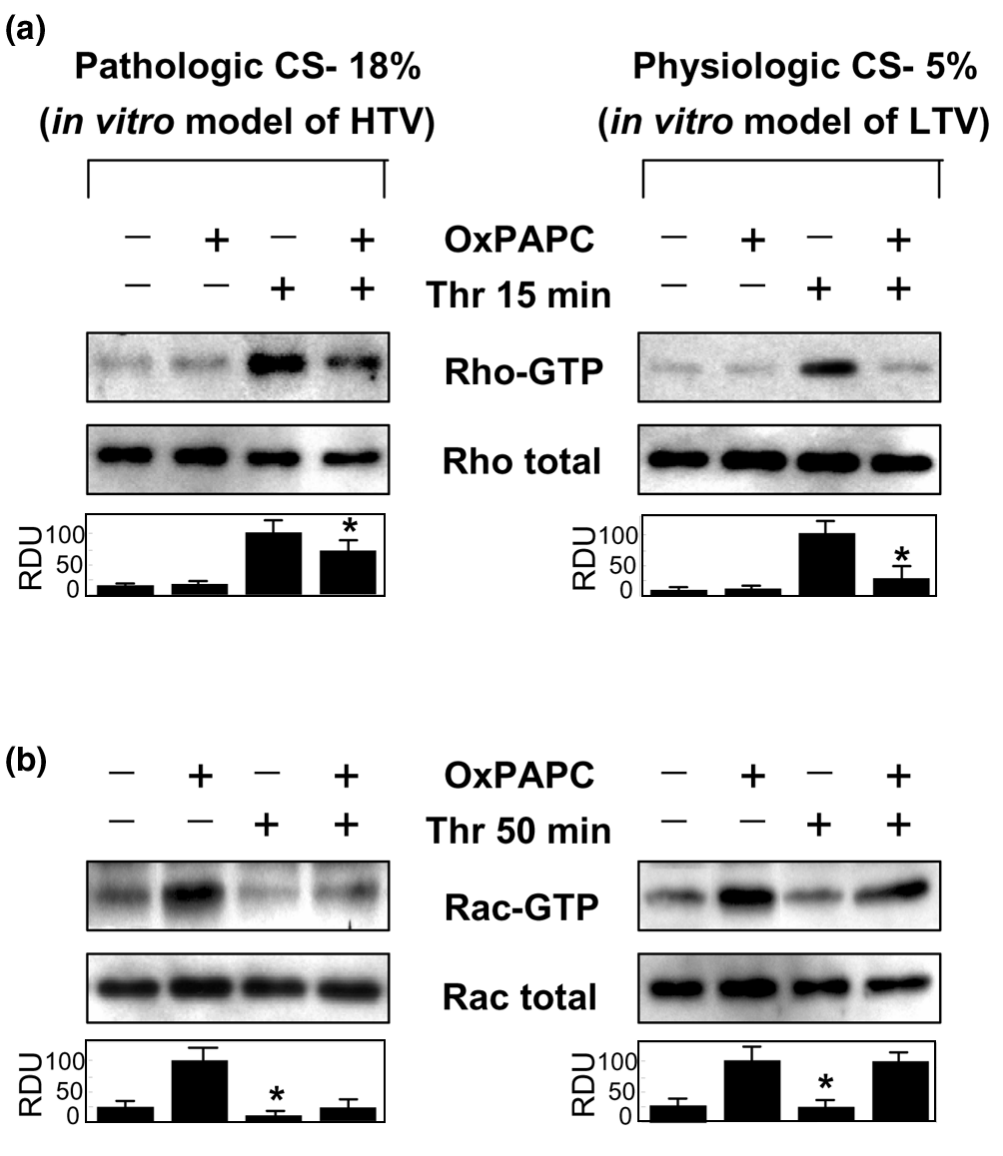
Effects of OxPAPC on RhoGTPase and RacGTPase activation in an *in vitro *model of ventilator-induced lung injury. Human pulmonary endothelial cells (ECs) were exposed to 5% cyclic stretch (CS) or 18% CS for 2 hours and pretreated with OxPAPC (20 μg/mL, 15 minutes) or vehicle prior to thrombin stimulation (0.5 U/mL, 15 or 50 minutes). Measurements of Rho **(a) **and Rac **(b) **activation were performed using pull-down assays as described in Materials and methods. In ECs exposed to 5% CS or 18% CS, OxPAPC attenuated thrombin-induced Rho activation during the acute phase (15 minutes) **(a) **and promoted Rac activation during the recovery phase (50 minutes) **(b) **after thrombin challenge. Graphs represent results of scanning densitometry of the membranes and are expressed in relative density units (RDUs). Results are mean ± standard deviation of three to five independent experiments. *p < 0.05, comparison between OxPAPC+Thr and Thr alone. HTV, high tidal volume; LTV, low tidal volume OxPAPC, oxidized 1-palmitoyl-2-arachidonoyl-sn-glycero-3-phosphorylcholine; Thr, thrombin.

The small GTPases Rho and Rac play opposing roles in the regulation of EC permeability [24,43,44]. Time-dependent effects of thrombin and OxPAPC on Rho and Rac activities in static EC cultures have been previously reported in our [21,22,33] and other studies. Similar to HPAEC cultures, direct activation of Rac by OxPAPC and OxPAPC-mediated attenuation of thrombin-induced Rho activity has also been observed in the lung microvascular EC cultures (data not shown). Thrombin-induced regulation of Rho and Rac in CS-stimulated pulmonary EC cultures has also been described in detail in our previous publications [23]. Our data demonstrated that, in comparison with static conditions, pathologic strain promoted thrombin-induced Rho activation during the acute phase and suppressed Rac activation during the recovery phase of thrombin-induced EC barrier disruption, whereas physiologic CS caused opposite effects on Rho and Rac. In the present study, Rho and Rac activities were measured *in vitro *at time points corresponding to the acute phase of thrombin-induced barrier disruption (5 to 15 minutes) and the recovery phase (50 minutes) based on our previous studies [23]. Pulmonary ECs were exposed to 5% CS or 18% CS for 2 hours and pretreated with OxPAPC (20 μg/mL, 15 minutes) or vehicle prior to thrombin stimulation (0.5 U/mL, 15 minutes). Thrombin-induced Rho activation was significantly increased in ECs preconditioned at 18% CS as compared with ECs exposed to 5% CS (Figure 8a). Furthermore, OxPAPC pretreatment significantly decreased thrombin-induced Rho activation in ECs exposed to both physiologic and pathologic CS levels (Figure 8a). Thus, the OxPAPC-induced attenuation of Rho activation in ECs exposed to 18% CS and thrombin observed in these experiments is highly consistent with the OxPAPC-induced reduction in actin stress fibers and paracellular gap formation described above (Figure 6).

We have previously shown that thrombin stimulation causes a decrease in basal Rac activity, which correlates with reciprocal Rho activation and Rho-dependent paracellular gap formation during the acute phase (15 minutes) of thrombin-induced EC barrier dysfunction. Subsequent increases in Rac activation were seen at later time points and corresponded to EC barrier recovery [22,23]. In this study, we analyzed Rac activation after 50 minutes of thrombin challenge in CS-preconditioned cells pretreated with OxPAPC or vehicle control. As above, pulmonary ECs were exposed to 5% CS or 18% CS for 2 hours and pretreated with OxPAPC (20 μg/mL, 15 minutes) or vehicle prior to thrombin stimulation (0.5 U/mL, 50 minutes). OxPAPC treatment of ECs exposed to 5% CS or 18% CS caused significant increases in Rac activity (Figure 8b). In ECs preconditioned at 5% CS, pretreatment with OxPAPC dramatically enhanced Rac activation during the recovery phase after 50 minutes of thrombin stimulation (Figure 8b, right panel), whereas in ECs exposed to 18% CS, Rac activity after 50 minutes of thrombin challenge was markedly lower and comparable between OxPAPC-pretreated and untreated cells. (Figure 8b, left panel). These results strongly suggest potential synergistic effects of OxPAPC and physiologic CS on Rac-mediated pulmonary EC monolayer recovery after challenge with edemagenic agents.

## Discussion

Vascular barrier dysfunction and acute lung inflammation are fundamental features that contribute to the significant mortality associated with VILI and ARDS. Despite advances in protective LTV ventilation strategies, effective pharmacotherapy for this devastating syndrome is lacking. Using an aseptic *in vivo *model of VILI, we show here for the first time that a single intravenous dose of OxPAPC significantly attenuates the early vascular barrier disruption and acute inflammation induced by mechanical ventilation at HTV. Intravenous OxPAPC significantly reduced alveolar and tissue inflammatory cell recruitment and protein accumulation in the BAL after 2 hours of mechanical ventilation at HTV.

In our previous study, we described similar protective effects of OxPAPC in an animal model of LPS-induced lung injury [20]. In that model, OxPAPC prevented neutrophil influx and barrier disruption likely in part via direct competitive inhibition of Toll-like receptor (TLR) binding [13,19,20]. However, despite the apparent similarities between VILI and LPS-induced lung injury, there are fundamental differences in the mechanisms leading to these pathologies. LPS-induced lung injury involves TLR-4-receptor-mediated activation of nuclear factor-kappa-B (NF-κB) and other pathways leading to an innate immune response, robust neutrophil infiltration, and lung tissue inflammation, which culminate in lung barrier dysfunction, edema, and compromised gas exchange. In turn, VILI induces a more modest acute inflammatory response with mild lung neutrophil accumulation and distinct mechanisms leading to inflammation and barrier dysfunction involving a different set of signaling molecules [23,45-47] and transcription factors [48]. For example, a group of genes upregulated by HTV mechanical ventilation alone (*ETF*, *E2F*, *Nrf1*, *CREB*, and *HIF1*) is not found in the list of genes upregulated by LPS [49]. In turn, LPS-induced genes (*ISRE*, *cREL*, *IRF*, *NF-κ B*, *ICSBP*, and *PU.1*) detected in the lung tissue are not upregulated by mechanical ventilation [49,50].

Clinical studies suggest that the vascular leak observed in VILI patients is caused by a combination of lung mechanical strain and increased levels of edemagenic and inflammatory mediators such as thrombin, histamine, tumor necrosis factor-alpha, and interleukin (IL)-8 and IL-1 [51-54]. Several two-hit animal models have been proposed to reproduce the VILI syndrome, combining experimentally induced lung inflammation (LPS, acid aspiration) and mechanical ventilation at HTVs to more appropriately reflect common comorbidities and risk factors present in patients with ALI [55]. In line with these observations, we used an *in vitro *model of pulmonary ECs preconditioned at a pathologically relevant level of CS (18% CS) [29,31,32] and stimulated with the edemagenic agonist thrombin to recapitulate VILI conditions *in vitro*.

Previous studies have shown that the EC barrier dysfunction induced by pathologic CS and thrombin is mediated by Rho-dependent mechanisms, whereas restoration of monolayer integrity is dependent on Rac activation [23]. Furthermore, Rac-dependent enhancement of peripheral actin cytoskeleton and EC barrier integrity has been observed in static EC cultures upon stimulation with OxPAPC [21,56]. Using an *in vitro *model of VILI, we demonstrated the protective effects of OxPAPC on barrier disruption induced by high-magnitude (18%) CS and thrombin and linked them with OxPAPC-induced reduction in Rho activity and modest increases in Rac activity. Furthermore, the combination of physiologically relevant CS (5% CS) and OxPAPC dramatically attenuated thrombin-induced Rho activation in the acute phase of EC barrier disruption and further promoted Rac activation associated with EC recovery after thrombin challenge.

The *in vitro *and *in vivo *results presented here, as well as our previously published results [20-22], strongly suggest that OxPAPC can prevent or reverse the increased endothelial permeability caused by a variety of barrier-disruptive agents, including inflammatory cytokines, edemagenic peptides, LPS, and high-magnitude CS. Furthermore, results from our *in vivo *model of VILI induced by HTV and TRAP-6 and data from pulmonary ECs exposed to pathologic CS and thrombin *in vitro *show potent barrier-protective effects of OxPAPC in the two-hit models of ALI. Taken together, these results strongly suggest that OxPAPC does not act as a specific anti-thrombin but rather promotes endothelial barrier function via regulation of Rac/Rho signaling leading to acceleration of EC monolayer recovery and enhancement of peripheral cytoskeleton and cell-cell junctions)[22,56-58]. Based on ample evidence from *in vitro *and *in vivo *studies, we believe that intravascular OxPAPC can act directly on the vascular endothelial layer and cause cytoskeletal changes that serve to counteract a variety of injurious insults that lead to barrier disruption.

Studies by several groups clearly suggest reciprocal relations between Rho and Rac activation [59-62] and opposing roles of Rho and Rac in maintaining EC barrier function [22,33,43,63]. Upstream mechanisms of Rho regulation and crosstalk between small GTPases Rho and Rac are the focus of current studies by several groups [23,64,65]. The results of this study show involvement of Rho-Rho kinase pathway in the development of lung vascular leak in response to HTV mechanical ventilation of mice, as vascular leak was significantly attenuated by pretreatment with Rho kinase inhibitor Y27632 (Figure 5). Our results also suggest suppression of agonist-induced activation of Rho pathway by oxidized phospholipids in pulmonary ECs (Figure 8).

Several potential mechanisms may be involved in the barrier-protective effects of and suppression of Rho signaling by OxPAPC. OxPAPC stimulates protein kinase A [16,66], which may suppress Rho activation via phosphorylation of Rho GDP dissociation inhibitor (RhoGDI) [67]. Other mechanisms may involve modulation of Rho-specific guanosine nucleotide exchange factors (GEFs) by signal protein kinases (PKA, PKC, and Src) activated by OxPAPC [66,68]. Recent studies suggest downregulation of the Rho pathway by Rac-mediated signaling cascades [60] via (a) direct Rac interaction with RhoGDI [59], (b) PAK1-dependent inhibition of Rho-specific GEF p115RhoGEF, and (c) stimulation of Rho-specific GTPase-activating protein p190-RhoGAP by Rac [61]. However, mechanisms of Rac-Rho crosstalk, though critical for endothelial permeability responses [21,23,69,70], are still poorly understood. Ongoing studies in our group are aimed to define upstream mechanisms of Rac activation by oxidized phospholipids and crosstalk between Rac and Rho signaling in the lung endothelial barrier regulation.

There is an apparent controversy between the protective effects of OxPAPC described in this study and the role of oxidant stress, lipid peroxidation, and tissue damage in patients with ARDS and ALI. Isoprostanes, prostanoid compounds primarily formed by non-enzymatic lipid peroxidation, have been used as markers of *in vivo *oxidant stress, and their plasma levels inversely correlate with outcome in patients with ARDS [71]. Pathologic oxidation of surfactant lipids results in the generation of both fragmented and oxygenated lipid peroxidation products, which may exert different effects on the alveolar epithelium [13,72]. Analysis of lipid peroxidation products associated with these pathologies indicates generation of fragmented phospholipids (such as POPVC and PGPC), which exhibit barrier-disruptive effects shown in our studies and other studies [21,73-75]. In contrast, *sn*-2-oxygenated, but not *sn*-2-fragmented, phospholipids (PEIPC and PECPC) are responsible for the OxPAPC-mediated Rac/Cdc42 activation, cytoskeletal remodeling, and induction of barrier-protective effects in the vascular endothelium [21]. Thus, selection for the protective *sn*-2-oxygenated products by precisely monitored oxidation of synthetic phospholipids or direct synthesis of lead compounds will be a promising but challenging task in generating a novel group of phospholipid-derived compounds combining anti-inflammatory and barrier-protective properties. In conclusion, this study demonstrates for the first time the protective effect of OxPAPC in the *in vivo *and *in vitro *models of VILI. Although further studies are needed to clarify molecular mechanisms of OxPAPC barrier-protective effects, these findings suggest that oxidized phospholipids and OxPAPC, in particular, may be considered as a new group of therapeutic candidates in ALI/VILI, combining anti-inflammatory and barrier-enhancing properties in the treatment of this devastating disease.

## Conclusion

The results presented here, as well as our previously published data [20-22,58], strongly suggest that OxPAPC can prevent or reverse the increased endothelial permeability caused by a variety of barrier-disruptive agents, including inflammatory cytokines, edemagenic peptides, LPS, and high-magnitude CS. Using the *in vivo *and *in vitro *models of VILI, we demonstrated for the first time the protective effect of oxidized phospholipids against early vascular barrier disruption and acute inflammation induced by mechanical ventilation at HTV. In contrast to the direct effects of OxPAPC on inhibition of the TLR-4-mediated inflammatory cascade in the model of LPS-induced lung inflammation and vascular dysfunction, we attribute the protective effects of OxPAPC on the lung vascular endothelium in the aseptic VILI model to the OxPAPC-induced activation of Rac signaling and reduction of Rho-induced endothelial hyperpermeability. These findings suggest that oxidized phospholipids may be considered as a new group of therapeutic candidates in ALI/VILI, combining anti-inflammatory and barrier-enhancing properties in the treatment of this devastating syndrome.

## Key messages

• Oxidized 1-palmitoyl-2-arachidonoyl-sn-glycero-3-phosphorylcholine (OxPAPC) significantly attenuates vascular leak, neutrophil accumulation, and Evans blue extravasation into the lung parenchyma caused by mechanical ventilation at high tidal volume and thrombin-derived signaling peptide TRAP-6.

• Protective effects of OxPAPC against VILI (ventilator-induced lung injury)-associated lung vascular leak have been reproduced in the pulmonary endothelial cell monolayers exposed to high-magnitude cyclic stretch and thrombin stimulation.

• Protection of endothelial monolayer integrity was due to OxPAPC-mediated attenuation of Rho pathway of endothelial barrier dysfunction.

## Abbreviations

ALI = acute lung injury; ARDS = acute respiratory distress syndrome; BAL = bronchoalveolar lavage; CS = cyclic stretch; DMPC = di-myristoyl-sn-glycero-3-phosphorylcholine; EC = endothelial cell; GEF = guanosine nucleotide exchange factor; HPAEC = human pulmonary artery endothelial cell; HPF = high power microscopic field; HTV = high tidal volume; IL = interleukin; LPS = lipopolysaccharide; LTV = low tidal volume; NF-κB = nuclear factor-kappa-B; OxPAPC = oxidized 1-palmitoyl-2-arachidonoyl-sn-glycero-3-phosphorylcholine; PAPC = 1-palmitoyl-2-arachidonoyl-sn-glycero-3-phosphorylcholine; PBS = phosphate-buffered saline; PEEP = positive end-expiratory pressure; PMN = polymorphonuclear leukocyte; RhoGDI = Rho GDP dissociation inhibitor; TLR = Toll-like receptor; TRAP-6 = thrombin receptor activating peptide-6; VILI = ventilator-induced lung injury.

## Competing interests

The authors declare that they have no competing interests.

## Authors' contributions

SN performed animal experiments, analyzed results, and was involved in manuscript preparation. PF and JX performed animal experiments. AAB performed immunofluorescence analysis and was involved in the discussion of the results. SC performed biochemical studies. VNB and NL synthesized the OxPAPC and participated in the manuscript discussion. JGNG funded *in vivo *studies. KGB initiated, reviewed, and coordinated the study and funded *in vivo *and *in vitro *experiments. All authors read and approved the final manuscript.

## References

[B1] Rubenfeld GD, Caldwell E, Peabody E, Weaver J, Martin DP, Neff M, Stern EJ, Hudson LD (2005). Incidence and outcomes of acute lung injury. N Engl J Med.

[B2] Ware LB, Matthay MA (2000). The acute respiratory distress syndrome. N Engl J Med.

[B3] Ranieri VM, Suter PM, Tortorella C, De Tullio R, Dayer JM, Brienza A, Bruno F, Slutsky AS (1999). Effect of mechanical ventilation on inflammatory mediators in patients with acute respiratory distress syndrome: a randomized controlled trial. JAMA.

[B4] Tremblay L, Valenza F, Ribeiro SP, Li J, Slutsky AS (1997). Injurious ventilatory strategies increase cytokines and c-fos m-RNA expression in an isolated rat lung model. J Clin Invest.

[B5] Villar J, Flores C, Méndez-Alvarez S (2003). Genetic susceptibility to acute lung injury. Crit Care Med.

[B6] Goodman RB, Pugin J, Lee JS, Matthay MA (2003). Cytokine-mediated inflammation in acute lung injury. Cytokine Growth Factor Rev.

[B7] Adhikari N, Burns KE, Meade MO (2004). Pharmacologic treatments for acute respiratory distress syndrome and acute lung injury: systematic review and meta-analysis. Treat Respir Med.

[B8] Pennathur S, Bergt C, Shao B, Byun J, Kassim SY, Singh P, Green PS, McDonald TO, Brunzell J, Chait A, Oram JF, O'brien K, Geary RL, Heinecke JW (2004). Human atherosclerotic intima and blood of patients with established coronary artery disease contain high density lipoprotein damaged by reactive nitrogen species. J Biol Chem.

[B9] Kalyanaraman B (2004). Nitrated lipids: a class of cell-signaling molecules. Proc Natl Acad Sci USA.

[B10] Morrow JD, Roberts LJ (2002). The isoprostanes: their role as an index of oxidant stress status in human pulmonary disease. Am J Respir Crit Care Med.

[B11] Waters CM (2004). Reactive oxygen species in mechanotransduction. Am J Physiol Lung Cell Mol Physiol.

[B12] Hammerschmidt S, Schiller J, Kuhn H, Meybaum M, Gessner C, Sandvoss T, Arnold K, Wirtz H (2003). Influence of tidal volume on pulmonary NO release, tissue lipid peroxidation and surfactant phospholipids. Biochim Biophys Acta.

[B13] Bochkov VN, Leitinger N, Birukov KG (2006). Role of oxidized phospholipids in acute lung injury. Curr Resp Med Rev.

[B14] Bochkov VN, Mechtcheriakova D, Lucerna M, Huber J, Malli R, Graier WF, Hofer E, Binder BR, Leitinger N (2002). Oxidized phospholipids stimulate tissue factor expression in human endothelial cells via activation of ERK/EGR-1 and Ca(++)/NFAT. Blood.

[B15] Lusis AJ (2000). Atherosclerosis. Nature.

[B16] Leitinger N, Tyner TR, Oslund L, Rizza C, Subbanagounder G, Lee H, Shih PT, Mackman N, Tigyi G, Territo MC, Berliner JA, Vora DK (1999). Structurally similar oxidized phospholipids differentially regulate endothelial binding of monocytes and neutrophils. Proc Natl Acad Sci USA.

[B17] Cole AL, Subbanagounder G, Mukhopadhyay S, Berliner JA, Vora DK (2003). Oxidized phospholipid-induced endothelial cell/monocyte interaction is mediated by a cAMP-dependent R-Ras/PI3-kinase pathway. Arterioscler Thromb Vasc Biol.

[B18] Ma Z, Li J, Yang L, Mu Y, Xie W, Pitt B, Li S (2004). Inhibition of LPS- and CpG DNA-induced TNF-alpha response by oxidized phospholipids. Am J Physiol Lung Cell Mol Physiol.

[B19] Bochkov VN, Kadl A, Huber J, Gruber F, Binder BR, Leitinger N (2002). Protective role of phospholipid oxidation products in endotoxin-induced tissue damage. Nature.

[B20] Nonas SA, Miller I, Kawkitinarong K, Chatchavalvanich S, Gorshkova I, Bochkov VN, Leitinger N, Natarajan V, Garcia JG, Birukov KG (2006). Oxidized phospholipids reduce vascular leak and inflammation in rat model of acute lung injury. Am J Respir Crit Care Med.

[B21] Birukov KG, Bochkov VN, Birukova AA, Kawkitinarong K, Rios A, Leitner A, Verin AD, Bokoch GM, Leitinger N, Garcia JG (2004). Epoxycyclopentenone-containing oxidized phospholipids restore endothelial barrier function via Cdc42 and Rac. Circ Res.

[B22] Birukova AA, Fu P, Chatchavalvanich S, Burdette D, Oskolkova O, Bochkov VN, Birukov KG (2007). Polar head groups are important for barrier protective effects of oxidized phospholipids on pulmonary endothelium. Am J Physiol Lung Cell Mol Physiol.

[B23] Birukova AA, Chatchavalvanich S, Rios A, Kawkitinarong K, Garcia JG, Birukov KG (2006). Differential regulation of pulmonary endothelial monolayer integrity by varying degrees of cyclic stretch. Am J Pathol.

[B24] Mehta D, Malik AB (2006). Signaling mechanisms regulating endothelial permeability. Physiol Rev.

[B25] Birukova AA, Zagranichnaya T, Alekseeva E, Fu P, Chen W, Jacobson JR, Birukov KG (2007). Prostaglandins PGE2 and PGI2 promote endothelial barrier enhancement via PKA- and Epac1/Rap1-dependent Rac activation. Exp Cell Res.

[B26] Bai KJ, Spicer AP, Mascarenhas MM, Yu L, Ochoa CD, Garg HG, Quinn DA (2005). The role of hyaluronan synthase 3 in ventilator-induced lung injury. Am J Respir Crit Care Med.

[B27] Moitra J, Sammani S, Garcia JG (2007). Re-evaluation of Evans Blue dye as a marker of albumin clearance in murine models of acute lung injury. Transl Res.

[B28] Watson AD, Leitinger N, Navab M, Faull KF, Hörkkö S, Witztum JL, Palinski W, Schwenke D, Salomon RG, Sha W, Subbanagounder G, Fogelman AM, Berliner JA (1997). Structural identification by mass spectrometry of oxidized phospholipids in minimally oxidized low density lipoprotein that induce monocyte/endothelial interactions and evidence for their presence *in vivo*. J Biol Chem.

[B29] Birukov KG, Jacobson JR, Flores AA, Ye SQ, Birukova AA, Verin AD, Garcia JG (2003). Magnitude-dependent regulation of pulmonary endothelial cell barrier function by cyclic stretch. Am J Physiol Lung Cell Mol Physiol.

[B30] Shikata Y, Rios A, Kawkitinarong K, DePaola N, Garcia JG, Birukov KG (2005). Differential effects of shear stress and cyclic stretch on focal adhesion remodeling, site-specific FAK phosphorylation, and small GTPases in human lung endothelial cells. Exp Cell Res.

[B31] Tschumperlin DJ, Oswari J, Margulies AS (2000). Deformation-induced injury of alveolar epithelial cells. Effect of frequency, duration, and amplitude. Am J Respir Crit Care Med.

[B32] Tschumperlin DJ, Margulies SS (1999). Alveolar epithelial surface area-volume relationship in isolated rat lungs. J Appl Physiol.

[B33] Birukova AA, Smurova K, Birukov KG, Kaibuchi K, Garcia JGN, Verin AD (2004). Role of Rho GTPases in thrombin-induced lung vascular endothelial cells barrier dysfunction. Microvasc Res.

[B34] Birukova AA, Adyshev D, Gorshkov B, Bokoch GM, Birukov KG, Verin AA (2006). GEF-H1 is involved in agonist-induced human pulmonary endothelial barrier dysfunction. Am J Physiol Lung Cell Mol Physiol.

[B35] Choi WI, Quinn DA, Park KM, Moufarrej RK, Jafari B, Syrkina O, Bonventre JV, Hales CA (2003). Systemic microvascular leak in an *in vivo *rat model of ventilator-induced lung injury. Am J Respir Crit Care Med.

[B36] Nonas SA, Miller IL, Garcia JG (2005). Strain-specific differences in vascular permeability following high volume mechanical ventilation in an *in vivo *rat model of acute lung injury. Proc Am Thorac Soc.

[B37] Quinn DA, Moufarrej RK, Volokhov A, Hales CA (2002). Interactions of lung stretch, hyperoxia, and MIP-2 production in ventilator-induced lung injury. J Appl Physiol.

[B38] Schultz MJ, Haitsma JJ, Zhang H, Slutsky AS (2006). Pulmonary coagulopathy as a new target in therapeutic studies of acute lung injury or pneumonia – a review. Crit Care Med.

[B39] Essler M, Amano M, Kruse HJ, Kaibuchi K, Weber PC, Aepfelbacher M (1998). Thrombin inactivates myosin light chain phosphatase via Rho and its target Rho kinase in human endothelial cells. J Biol Chem.

[B40] Clements RT, Minnear FL, Singer HA, Keller RS, Vincent PA (2005). RhoA and Rho-kinase dependent and independent signals mediate TGF-beta-induced pulmonary endothelial cytoskeletal reorganization and permeability. Am J Physiol Lung Cell Mol Physiol.

[B41] Birukova AA, Birukov KG, Adyshev D, Usatyuk P, Natarajan V, Garcia JG, Verin AD (2005). Involvement of microtubules and Rho pathway in TGF-beta1-induced lung vascular barrier dysfunction. J Cell Physiol.

[B42] Sun H, Breslin JW, Zhu J, Yuan SY, Wu MH (2006). Rho and ROCK signaling in VEGF-induced microvascular endothelial hyperpermeability. Microcirculation.

[B43] Wojciak-Stothard B, Ridley AJ (2002). Rho GTPases and the regulation of endothelial permeability. Vascul Pharmacol.

[B44] Dudek SM, Garcia JG (2001). Cytoskeletal regulation of pulmonary vascular permeability. J Appl Physiol.

[B45] Chapman KE, Sinclair SE, Zhuang D, Hassid A, Desai LP, Waters CM (2005). Cyclic mechanical strain increases reactive oxygen species production in pulmonary epithelial cells. Am J Physiol Lung Cell Mol Physiol.

[B46] Frank JA, Matthay MA (2003). Science review: mechanisms of ventilator-induced injury. Crit Care.

[B47] Frank JA, Pittet JF, Lee H, Godzich M, Matthay MA (2003). High tidal volume ventilation induces NOS2 and impairs cAMP-dependent air space fluid clearance. Am J Physiol Lung Cell Mol Physiol.

[B48] Wurfel MM (2007). Microarray-based analysis of ventilator-induced lung injury. Proc Am Thorac Soc.

[B49] Gharib SA, Liles WC, Matute-Bello G, Glenny RW, Martin TR, Altemeier WA (2006). Computational identification of key biological modules and transcription factors in acute lung injury. Am J Respir Crit Care Med.

[B50] Elkon R, Linhart C, Sharan R, Shamir R, Shiloh Y (2003). Genome-wide in silico identification of transcriptional regulators controlling the cell cycle in human cells. Genome Res.

[B51] Narimanbekov IO, Rozycki HJ (1995). Effect of IL-1 blockade on inflammatory manifestations of acute ventilator-induced lung injury in a rabbit model. Exp Lung Res.

[B52] Pugin J, Dunn I, Jolliet P, Tassaux D, Magnenat JL, Nicod LP, Chevrolet JC (1998). Activation of human macrophages by mechanical ventilation *in vitro*. Am J Physiol.

[B53] Vlahakis NE, Schroeder MA, Limper AH, Hubmayr RD (1999). Stretch induces cytokine release by alveolar epithelial cells *in vitro*. Am J Physiol.

[B54] Dos Santos CC, Slutsky AS (2000). Invited review: mechanisms of ventilator-induced lung injury: a perspective. J Appl Physiol.

[B55] Matthay MA, Zimmerman GA, Esmon C, Bhattacharya J, Coller B, Doerschuk CM, Floros J, Gimbrone MA, Hoffman E, Hubmayr RD, Leppert M, Matalon S, Munford R, Parsons P, Slutsky AS, Tracey KJ, Ward P, Gail DB, Harabin AL (2003). Future research directions in acute lung injury: summary of a National Heart, Lung, and Blood Institute working group. Am J Respir Crit Care Med.

[B56] Birukova AA, Malyukova I, Mikaelyan A, Fu P, Birukov KG (2007). Tiam1 and betaPIX mediate Rac-dependent endothelial barrier protective response to oxidized phospholipids. J Cell Physiol.

[B57] Birukova AA, Malyukova I, Poroyko V, Birukov KG (2007). Paxillin – {beta}-catenin interactions are involved in Rac/Cdc42-mediated endothelial barrier-protective response to oxidized phospholipids. Am J Physiol Lung Cell Mol Physiol.

[B58] Birukova AA, Chatchavalvanich S, Oskolkova O, Bochkov VN, Birukov KG (2007). Signaling pathways involved in OxPAPC-induced pulmonary endothelial barrier protection. Microvasc Res.

[B59] Wong KW, Mohammadi S, Isberg RR (2006). Disruption of RhoGDI and RhoA regulation by a Rac1 specificity switch mutant. J Biol Chem.

[B60] Rosenfeldt H, Castellone MD, Randazzo PA, Gutkind JS (2006). Rac inhibits thrombin-induced Rho activation: evidence of a Pak-dependent GTPase crosstalk. J Mol Signal.

[B61] Herbrand U, Ahmadian MR (2006). p190-RhoGAP as an integral component of the Tiam1/Rac1-induced downregulation of Rho. Biol Chem.

[B62] Klinger JR, Warburton R, Carino GP, Murray J, Murphy C, Napier M, Harrington EO (2006). Natriuretic peptides differentially attenuate thrombin-induced barrier dysfunction in pulmonary microvascular endothelial cells. Exp Cell Res.

[B63] Birukova AA, Alekseeva E, Mikaelyan A, Birukov KG (2007). HGF attenuates thrombin-induced permeability in the human pulmonary endothelial cells by Tiam1-mediated activation of the Rac pathway and by Tiam1/Rac-dependent inhibition of the Rho pathway. FASEB J.

[B64] DerMardirossian C, Schnelzer A, Bokoch GM (2004). Phosphorylation of RhoGDI by Pak1 mediates dissociation of Rac GTPase. Mol Cell.

[B65] Tamma G, Klussmann E, Procino G, Svelto M, Rosenthal W, Valenti G (2003). cAMP-induced AQP2 translocation is associated with RhoA inhibition through RhoA phosphorylation and interaction with RhoGDI. J Cell Sci.

[B66] Birukov KG, Leitinger N, Bochkov VN, Garcia JG (2004). Signal transduction pathways activated in human pulmonary endothelial cells by OxPAPC, a bioactive component of oxidized lipoproteins. Microvasc Res.

[B67] Qiao J, Huang F, Lum H (2003). PKA inhibits RhoA activation: a protection mechanism against endothelial barrier dysfunction. Am J Physiol Lung Cell Mol Physiol.

[B68] Zheng Y (2001). Dbl family guanine nucleotide exchange factors. Trends Biochem Sci.

[B69] Kouklis P, Konstantoulaki M, Vogel S, Broman M, Malik AB (2004). Cdc42 regulates the restoration of endothelial barrier function. Circ Res.

[B70] Shikata Y, Birukov KG, Birukova AA, Verin AD, Garcia JG (2003). Involvement of site-specific FAK phosphorylation in sphingosine-1 phosphate- and thrombin-induced focal adhesion remodeling: role of Src and GIT. FASEB J.

[B71] Carpenter CT, Price PV, Christman BW (1998). Exhaled breath condensate isoprostanes are elevated in patients with acute lung injury or ARDS. Chest.

[B72] Pulfer MK, Murphy RC (2004). Formation of biologically active oxysterols during ozonolysis of cholesterol present in lung surfactant. J Biol Chem.

[B73] Qiao J, Huang F, Naikawadi RP, Kim KS, Said T, Lum H (2006). Lysophosphatidylcholine impairs endothelial barrier function through the G protein-coupled receptor GPR4. Am J Physiol Lung Cell Mol Physiol.

[B74] Janssen LJ, Catalli A, Helli P (2005). The pulmonary biology of isoprostanes. Antioxid Redox Signal.

[B75] Milne GL, Musiek ES, Morrow JD (2005). The cyclopentenone (A2/J2) isoprostanes – unique, highly reactive products of arachidonate peroxidation. Antioxid Redox Signal.

